# Relationship between lipoprotein(a) and PCSK9 in angiogram-proven premature coronary artery disease in an Asian cohort

**DOI:** 10.1038/s41598-026-36716-7

**Published:** 2026-01-22

**Authors:** Rahayu Zulkapli, Suhaila Abd Muid, Seok Mui Wang, Ahmad Bakhtiar Md Radzi, Khairul Shafiq Ibrahim, Mohd Yusmiaidil Putera Mohd Yusof, Hapizah Nawawi

**Affiliations:** 1 Cardiovascular Advancement and Research Excellence Institute (CARE Institute), Universiti Teknologi MARA, Level 4, Academic Building, Jalan Hospital, 47000 Sungai Buloh, Selangor Malaysia; 2 Faculty of Medicine, Universiti Teknologi MARA (UiTM), Sungai Buloh Campus, Jalan Hospital, Sungai Buloh, 47000 Selangor Malaysia; 3 Faculty of Dentistry, Universiti Teknologi MARA (UiTM), Sungai Buloh Campus, Jalan Hospital, Sungai Buloh, 47000 Selangor Malaysia

**Keywords:** Premature coronary artery disease, Coronary artery disease, Familial hypercholesterolaemia, Proprotein convertase subtilisin/Kexin type 9, PCSK9, Lipoprotein (a), Biomarkers, Cardiology, Diseases, Medical research, Risk factors

## Abstract

Coronary artery disease (CAD) has been associated with elevated Lp(a) levels, yet the mechanism driving the pro-atherogenic and inflammatory effects remains unclear. Proprotein convertase subtilisin/kexin type 9 (PCSK9), a key regulator of lipid metabolism with emerging roles in vascular inflammation. This study explored the relationship between Lp (a) and PCSK9 in an Asian cohort with angiogram-proven premature CAD (AP-pCAD), with and without familial hypercholesterolemia (FH). Patients were recruited from Cardiology and Specialist Lipid Clinics; grouped into pCAD with FH (*n* = 70), pCAD without FH (*n* = 65), and normal controls (G3; *n* = 69). FH was clinically diagnosed using the Dutch Lipid Clinic Network. Lp(a) and PCSK9 levels were measured using an automated chemistry analyser and ELISA. Lp(a) and PCSK9 levels were significantly higher in pCAD groups compared to controls. No significant correlation between Lp(a) and PCSK9 was observed in individual pCAD subgroups (G1 or G2); a weak positive correlation was found in the normal control group (G3; *r* = 0.366, *p* = 0.019). In multivariate analysis, Lp(a) emerged as a significant independent predictor of pCAD (adjusted OR: 5.036, *p* = 0.015). In conclusion, Lp(a) independently predicts pCAD, while its association with PCSK9 appears modest and context-dependent, suggesting a more complex interplay possibly influenced by lipid-lowering therapy such as statin use.

## Introduction

 Atherosclerotic cardiovascular disease (ASCVD) in young adults represents a specific and growing challenge. Young people may be less aware of their underlying cardiovascular risk factors and are less likely to discuss lifestyle modification or primary prevention with their healthcare providers^1,2^. The burden of ASCVD in young adults is a significant public health issue because of the potential loss of lifetime productivity and increased lifetime healthcare use. Moreover, recent reports highlight that the myocardial infarction (MI) rate reduction has not extended to young adults but continues to have worse cardiovascular outcomes than men^3,4^. Premature coronary artery disease (pCAD) is defined as coronary artery disease (CAD) with age of onset below 55 in men and 60 in women^5^.

According to the European Society of Cardiology/European Atherosclerosis Society (ESC/EAS) and American College of Cardiology/American Heart Association (ACC/AHA) guidelines, lipoprotein (a) (Lp(a)) is a genetically predisposed lipoprotein known as an independent factor in the development of cardiovascular disease^6–10^. Elevated Lp(a), also called hyperlipoproteinemia(a), is relatively independent of age and gender and varies up to 1000-fold among individuals (including siblings). Diet and nutritional factors have minimal influence on Lp(a) levels^10^. Lp(a) adversely affects endothelial function, inflammation, oxidative stress, fibrinolysis, and plaque stability, leading to accelerated atherothrombosis and pCAD. Lp(a) not only contains all the proatherogenic components of low-density lipoprotein (LDL) but also of apolipoprotein (a) (apo(a)). According to Hovingh et al.^11^ and Duarte & Giugliano^12^, Lp(a) is linked to an increased risk of CAD, and Langston et al.^13^ found that a high concentration of Lp(a) is a common genetic risk factor for CAD and MI in the general population. Lp(a) levels have been significantly higher in familial hypercholesterolaemia (FH) patients, particularly those with an early CAD episode^14^. At the same time, Langsted et al. investigated the Lp(a) concentration in individuals with clinically diagnosed FH compared to those unlikely to have FH in Denmark’s general population. Their study highlighted higher Lp(a) concentrations in clinical FH without mutations^13^. In contrast, Perez et al.^15^ found that increased Lp(a) concentrations in FH patients were related to LDL receptor (LDLR) dysfunction, which affects LDL cholesterol (LDL-c) clearance. Lp(a) levels were found to vary among different populations^13,16^. Despite the divergent views, the underlying reason for higher Lp(a) concentrations in FH patients remains unknown.

In 2003, the discovery of PCSK9 marked an extraordinary paradigm shift, as it led to the development of a potent arsenal of inhibitory medicine that effectively lowered LDL-c to previously unattainable levels. Currently, a considerable number of drug candidates that impede the PCSK9 pathway have commenced preclinical or early-phase clinical trials. Notably, two of these drugs, Evolocumab and Alirocumab, were approved by the US FDA in 2015. According to Shapiro & Fazio, preclinical studies demonstrated that PCSK9 is capable of producing pleiotropic effects in addition to its modulation of plasma LDL levels. Before PCSK9 discovery, there were only two known genes (LDLR and ApoB)^17^ linked with FH in humans. The tremendous interest in PCSK9 was sparked by the findings that gain-of-function (GOF) mutations are associated with hypercholesterolaemia and increased coronary events, while loss-of-function (LOF) mutations lead to a reduction of circulating LDL and decreased risk of developing CAD. A GOF mutation in the PCSK9 gene was found to cause FH^18–22^.

PCSK9 gain-of-function (GOF) mutation causes hypercholesterolemia, while loss-of-function (LOF) mutations of PCSK9 can result in hypocholesterolemia^23,24^. It has been demonstrated that plasma PCSK9 levels are elevated in acute MI and are associated with the severity of CAD^25,26^. Several demographic and metabolic parameters appear to correlate with serum PCSK9 levels, including plasma LDL-c, high-density lipoprotein (HDL) cholesterol, triglycerides (TG), apolipoprotein B (apo B), insulin, glucose, smoking, and body mass index (BMI)^27–29^. Besides, the correlation between PCSK9 and Lp(a) is poorly established^30,31^. There were few studies concerning the association of PCSK9 with Lp(a) that have been reported, such as Tavori et al.^32^ found a relationship between PCSK9 and Lp(a), but a small sample size was used together with the absence of a control group. A recent study by Nekaies et al.^31^ reported that plasma PCSK9 levels correlated positively with Lp(a) concentrations in 50 patients with type 2 diabetes mellitus (T2DM) but not in non-T2DM subjects in Tunisians. Interestingly, they found no positive association of PCSK9 with total cholesterol (TC) or LDL-c, which is different from previous studies in both T2DM patients and non-T2DM subjects^33–37^. Moreover, both Lp(a) and PCSK9 levels considerably vary by race and other factors^29,37,38^.

However, to our knowledge, the relationship between Lp (a) and PCSK9 in the angioproven-pCAD (AP-pCAD) subjects with and without FH patients in the Asian cohort has not been investigated thus far. Therefore, this study was conducted to investigate the correlation and association between PCSK9 and Lp(a) level in patients with FH among pCAD patients. Thus, an investigation on the lipid profile, PCSK9 and Lp(a) levels in gender and age-matched subjects with and without CAD was conducted.

## Methods

### Study design

This cross-sectional study analyses various clinical and biochemical data from study subjects through population-based screening programs via convenient sampling. The community health screening programmes were conducted in East and West Malaysia, incorporating the various states and regions in the north, south, central, east, and west parts of the country to represent the whole population for the normal control (NC) group, group 3, non-CAD, and non-FH (CAD- FH-). The recruitment of patients in group 1, angiogram proven-pCAD (AP-pCAD) patients who were clinically identified as FH (pCAD + FH+), and group 2, AP-pCAD patients who were clinically identified as non-FH (pCAD + FH-), was performed in the National Heart Institute (IJN) and UiTM specialist clinics, including Cardiology and Specialist Lipid Clinics, based on the inclusion and exclusion criteria. This study also involved the recruitment of known and undiagnosed hypercholesterolemic individuals in a community setting. The diagnosis of FH was determined using DLCC. Healthy individuals with normal cholesterol levels without CAD or clinically diagnosed FH were recruited as normal controls.

### Ethical approval

Ethical approvals for this study have been obtained from UiTM Research Ethics Committee [REC/02/2023 (PG/MR/45)] and IJN Ethics Committee [IJNEC/03/2012 (6)] prior to the commencement of this study. The study was conducted in accordance with the Declaration of Helsinki. All subjects provided written informed consent before enrolment into the study.

### Inclusion criteria

The general inclusion criteria for group 1 (G1) and group 2 (G2) were Malaysian males and females aged ≥ 18 years old with angiogram-proven pCAD (AP-pCAD) and who voluntarily consented to participate in this study (Table 1). This study included group 3 (G3), healthy individuals with normal serum cholesterol levels and no known history of CAD or FH.

### Definition of pCAD for this study

Subjects with pCAD were defined as those with the first diagnosis of CAD at < 55 years old in males and < 60 years old in females^5^.

**Table 1:** Inclusion Criteria.

GROUP 1	GROUP 2	GROUP 3
pCAD (+) FH (+)	pCAD (+) FH (-)	CAD (-) FH (-)
Malaysians		
Not pregnant/Absence of physical disabilities		
Definite, Probable, or Possible FH by DLCC	Unlikely FH by DLCC	
Has undergone at least one of the listed procedures:		a) No past history or record of CAD
i) An abnormal coronary angiogram with stenosis of > 50% in at least one major epicardial artery segment^39^ and/or,		b) Has no previous history of undergoing any of the following procedures:
ii) Percutaneous coronary intervention (PCI) and/or,		i) Coronary angiogram (stenosis of > 50% in at least one major epicardial artery segment) and/or,
iii) Coronary artery bypass graft surgery (CABG)^40^		ii) Percutaneous coronary intervention (PCI) and/or,
		iii) Coronary artery bypass graft surgery (CABG).
Statin Treatment		Not on Statin Treatment

No secondary hypercholesterolemia.

### Exclusion criteria

The exclusion criteria for G1 and G2 were non-Malaysian males and females aged ≤ 18 years old, pregnant women, those without angiogram-confirmed PCAD diagnosis (in G1 and G2), history of PCI and CABG, and those with known causes of secondary hypercholesterolaemia were excluded (Table 1). Electronic hospital medical records were reviewed for clinical and medication history of known secondary hypercholesterolaemia, which includes hypothyroidism, nephrotic syndrome, and cholestasis. Control subjects were individuals without a history of CAD, selected from the community health screening programmes. Only Malaysian citizens who were age- and gender- matched were selected for this study. Non-Malaysian, those who were pregnant and with known secondary hypercholesterolaemia were excluded from the study. If any documentation, clinical indication, and/or abnormal blood test result were not present, the subjects were considered as not having any secondary hypercholesterolaemia. Those with secondary hypercholesterolaemia may have similar lipid profiles to those with FH. Besides, certain types of drugs may also contribute to the development of secondary hypercholesterolaemia.

### Clinical diagnosis of FH according to DLCC

All eligible subjects were classified into possible FH (3–5 points), probable FH (6–8 points), or definite FH (≥ 8 points) categories using modified DLCC criteria^41^. The Dutch Lipid Clinic Network (DLCC) criteria used in this study serve as the diagnostic method, which includes LDL-C levels and the following clinical characteristics:

i. Family history (1st degree relative: parent, sibling, child):

- 1 st degree relatives with known premature CAD (< 55 years in men, < 60 years in women).
- 1 st degree relatives with LDL-c > 4.9 mmol/L (95th percentile).
- 1 st degree relatives with tendon xanthoma AND/OR corneal arcus.
- Children (< 18 years) with LDL-c > 95th percentile for their age group.

ii. Personal clinical history:

- Premature CAD (men < 55, women < 60).
- Premature cerebral or peripheral vascular disease (men < 55, women < 60).

iii. Physical examination:

- Tendon xanthomata.
- Corneal arcus in individuals under 45 years.

iv. LDL-C level (mmol/L).

The measurement of LDL-c was then calculated from the measurements of TC, HDL, and TG values using the Friedewald equation^42^ as follows:

[TC (mmol/L)] – [HDL-c (mmol/L)] – [TG (mmol/L)/2.2] = [LDL-c (mmol/L)]

The Friedewald equation was verified by comparing its results with those of a reference method known as beta-quantification, where total LDL mass is directly measured by analytical ultracentrifugation^43^. Calculated LDL-c levels were used to reflect the baseline LDL-c level. Their plasma LDL-c was adjusted by a correction factor that depends on the type and dosage of specific statins to estimate pre-treatment LDL-c levels^44^.

### Subject recruitment

Written informed consent and a standard validated questionnaire^45^ were given to be filled in and signed by the subjects upon recruitment. The questionnaire used in this study includes personal information regarding the personal and family medical history, anthropometric data, and clinical findings of the subjects. Clinical data were obtained by clinicians who were part of the research project to ensure accurate clinical assessment of the subjects.

Demographic data obtained includes age, gender, ethnicity, gross household income, education, marital status, personal and family history of hypertension, hypercholesterolemia, CVD, and other lifestyle habits, such as smoking status, alcohol intake, measurements of body mass index (BMI), waist circumference (WC), hip circumference (HC), Waist-to-Hip Ratio (WHR), and blood pressure. The clinical examination includes examination for lipid stigmata, including the presence of corneal arcus, the skin of the eyelids (xanthelasma), and tendon xanthomas. Upon completion of the questionnaire and consent to participate, the questionnaires were collected, and the information was transferred to the database for further statistical analysis.

### Blood sample collection

Blood samples were collected into plain blood collection tubes in the morning after an overnight fast (of ≥ 8 h) and centrifuged at 4000 rpm for 20 min to obtain serum after being set upright for at least 30 min. The serum was transferred using a syringe pipette into a 1.5 mL microcentrifuge tube and was kept in a −20 °C freezer till analysis.

### Measurement of Lp(a)

Lp(a) was measured using an automated clinical chemistry analyser, Cobas Roche C501 (Roche Diagnostics), configured to nmol/L units. The principle of Lp(a) detection was turbidimetrically determined at wavelengths 800/660 nm.

### Measurement of total protein PCSK9

Concentrations of total PCSK9 in the blood serum were measured with a commercially available standard ELISA kit (Elabscience, Texas, United States), according to the manufacturer’s instructions. The absorbance was measured at 450 nm using a microplate reader (Agilent, California, United States). All procedures were performed according to the manufacturer’s instructions. The absorbance of the samples was measured at 450 nm using a microplate reader (Agilent, California, United States).

### Statistical analysis

Data were analyzed using IBM SPSS Statistics version 26 (IBM, NY, USA). data were presented as means (SD) for continuous variables (after checking the data distribution by performing the Kolmogorov-Smirnov test) and percentages for categorical variables. The common risk factor for pCAD was calculated by combining G1 and G2 [(YES categorical variables/total number of patients) ×100%]. The significance of differences between the numerical variables was determined by using a two-sample t-test and One-Way ANOVA (for parametric tests) or Mann-Whitney and Kruskal-Wallis tests (for non-parametric tests), while the significance of association between categorical variables was determined by using a Chi-squared test^46^. The association of Lp(a) and PCSK9 and the coronary risk factor was analysed using Spearman’s rank correlation. All final analyses with p-value < 0.05 were considered statistically significant. Logistic regression was used to determine the association of independent variables among pCAD patients after correcting for the confounding factors. Independent variables with a p-value < 0.25 were included for multiple logistic regression analysis. The ‘Enter’ method was used for the variable selection method. Two-way interactions of each coronary risk factor (CRF) were checked, and relevant significant variables were included in the model. The independent variables were included in a multiple logistic regression, and multicollinearity was checked. Hosmer Lemeshow Goodness of Fit test^47^, the classification table, and the receiver operator characteristic curve were performed to test the model’s fitness. All final analyses with p-value < 0.05 were considered statistically significant.

## Results

### Baseline characteristics and coronary risk factors of the study population

Table 2 shows the distribution of participants into groups based on the presence of pCAD and clinical diagnosis of FH according to the DLCC criteria. Malay was the major ethnic group across all groups. G1 and G2 (participants with pCAD) have higher proportions of obesity (BMI) and hypertension compared to G3, the normal control group (participants without CAD and FH) (*p* < 0.001). The presence of a family history of pCAD was also significantly higher in G1 compared to the other groups (*p* < 0.001). Similarly, there was a significantly higher percentage of individuals with a family history of HC in G1 compared to G2 and G3 (*p* < 0.001). Only two patients (2.9%) presented with tendon xanthoma (TX), and six patients (8.6%) with corneal arcus in G1. TC, TG, and LDL-c levels of G1 were significantly higher than G2 but significantly lower than G3. The median Lp(a) and PCSK9 level was highest in the pCAD group (G1 and G2), whilst it appeared lowest in the normal control group (G3) (Table 3).

**Table 2:** Distribution of individuals based on the presence of CAD and clinical diagnosis of FH (*n* = 204).

Parameters	GROUP 1	GROUP 2	GROUP 3	*p*-value
	pCAD (+) FH (+)	pCAD (+) FH (-)	CAD (-) FH (-)	
	*n* = 70	*n* = 65	*n* = 69	
Gender				
Male	59 (84.3)	57 (87.7)	47 (68.1)	NS ^
Female	11 (15.7)	8 (12.3)	22 (31.9)	
Age (years)	52.3 ± 6.9	53.1 ± 8.1	51.13 ± 2.8	NS @
Age range (years)	34–64	33–72	34–53	-
Ethnicity				< 0.05^
Malay	48 (68.6)	52 (80.0)	42 (59.4)	
Chinese	5 (7.1)	6 (9.2)	3 (2.9)	
Indian & Others	17 (24.3)	7 (10.8)	26 (37.7)	
BMI Categories				< 0.001^
Normal	11 (15.7)	23 (35.4)	11 (15.9)	
Overweight	5 (7.1)	15 (23.1)	9 (13.0)	
Obese	54 (77.2)	27 (41.5)	49 (71.0)	
Central Obesity				< 0.001^
Yes	50 (71.4)	28 (43.1)	50 (72.5)	
No	20 (28.6)	37 (56.9)	19 (27.5)	
Smoker				< 0.05^
Current smoker	19 (27.1)	9 (13.8)	16 (23.2)	
Ex-smoker	18 (25.7)	28 (43.1)	12 (17.4)	
Non-smoker	33 (47.2)	28 (43.1)	41 (59.4)	
Diabetes				< 0.001^
Yes	39 (55.7)	36 (55.44)	4 (5.8)	
No	31 (44.3)	29 (44.6)	65 (94.2)	
Hypertension				< 0.001^
Yes	46 (65.7)	40 (61.5)	18 (26.1)	
No	24 (34.3)	25 (38.5)	51(73.9)	
Hypercholesterolaemia				< 0.001^
Yes	70 (100.00)	0 (0.0)	40 (58.0)	
No	0 (0.0)	65 (100.0)	29 (42.0)	
Statin therapy				< 0.001^
Yes	70 (100.0)	65 (100.0)	0 (0.0)	
No	0 (0.0)	0 (0.0)	69 (100.0)	
Family history of pCAD				< 0.001^
Yes	27 (38.6)	0 (0.0)	5 (7.2)	
No	43 (61.4)	65 (100.0)	65 (92.8)	
Family history of HC				< 0.001^
Yes	38 (54.3)	14 (21.5)	9 (13.0)	
No	32 (45.7)	51 (78.5)	60 (87.0)	
Tendon xanthomata				NS^
Yes	2 (2.9)	0 (0.0)	0 (0.0)	
No	68 (97.1)	65 (100.0)	67 (100.0)	
Corneal arcus (< 45 years)				< 0.05^
Yes	6 (8.6)	0 (0.0)	0 (0.0)	
No	64 (91.4)	65 (100.0)	69 (100.0)	

Data are presented as numbers (n) and percentages (%) for categorical data, mean and standard deviation (SD) for continuous data. Statistical analysis: ^Chi-squared test, @ One-Way ANOVA test. Abbreviations: FH (familial hypercholesterolemia), pCAD (premature coronary artery disease), HC (hypercholesterolemia), BMI (body mass index).

**Table 3:** Lipid profile, Lp(a), and PCSK9 based on the presence of CAD and clinical diagnosis of FH (*n* = 204).

Parameters	GROUP 1	GROUP 2	GROUP 3	*p*-value
	pCAD (+) FH (+)	pCAD (+) FH (-)	CAD (-) FH (-)	
Lipid Profile at Study Entry				
Pre-treatment LDL-c (mmol/l) ^**, 1^	5.2 ± 1.8 ^a, b^	3.3 ± 0.8 ^a, b^	2.9 ± 0.8 ^a^	< 0.001*
Post-treatment LDL-c (mmol/l)	2.4 ± 0.8	1.7 ± 0.4	-	NS*
TC (mmol/l)	4.4 ± 1.0 ^a^	3.5 ± 0.7 ^a^	5.2 ± 1.1 ^a^	< 0.001*
TG (mmol/l)	1.6 ± 0.8 ^a, c^	1.5 ± 0.9 ^b, c^	2.4 ± 1.6 ^a, b, c^	< 0.001*
HDL-c (mmol/l)	1.2 ± 0.2	1.1 ± 0.2	1.0 ± 0.3	NS*
**Lp(a) (mmol/l)**	27.2 (13.2–72.2) ^a^	34.7 (12.7–100.9) ^b^	7.5(7.0–14.7) ^a, b^	< 0.001*
**PCSK9 (ng/ml)**	431.4 (178.0–1008.0) ^a^	471.4 (333.1–1188) ^b^	389.7(147.1–566.2) ^a, b^	< 0.05*

Data are presented as mean and standard deviation (SD) and median (interquartile range) [IQR] for continuous data.**Representing baseline LDL-c level prior to lipid-lowering medication and LDL-c level for drug-naïve individuals. 1 Several patients do not have the exact baseline lipid profile – statin conversion was used to calculate the estimated post-treatment LDL-c level. a, b, c = *p* < 0.05. Statistical tests with the same symbols in the same row are significantly different from each other. Abbreviation: LDL-c (low-density lipoprotein cholesterol), TC (total cholesterol), TG (triglyceride), HDL-c (high-density lipoprotein cholesterol).

### Conventional risk factors in pCAD patients

**Fig. 1:**
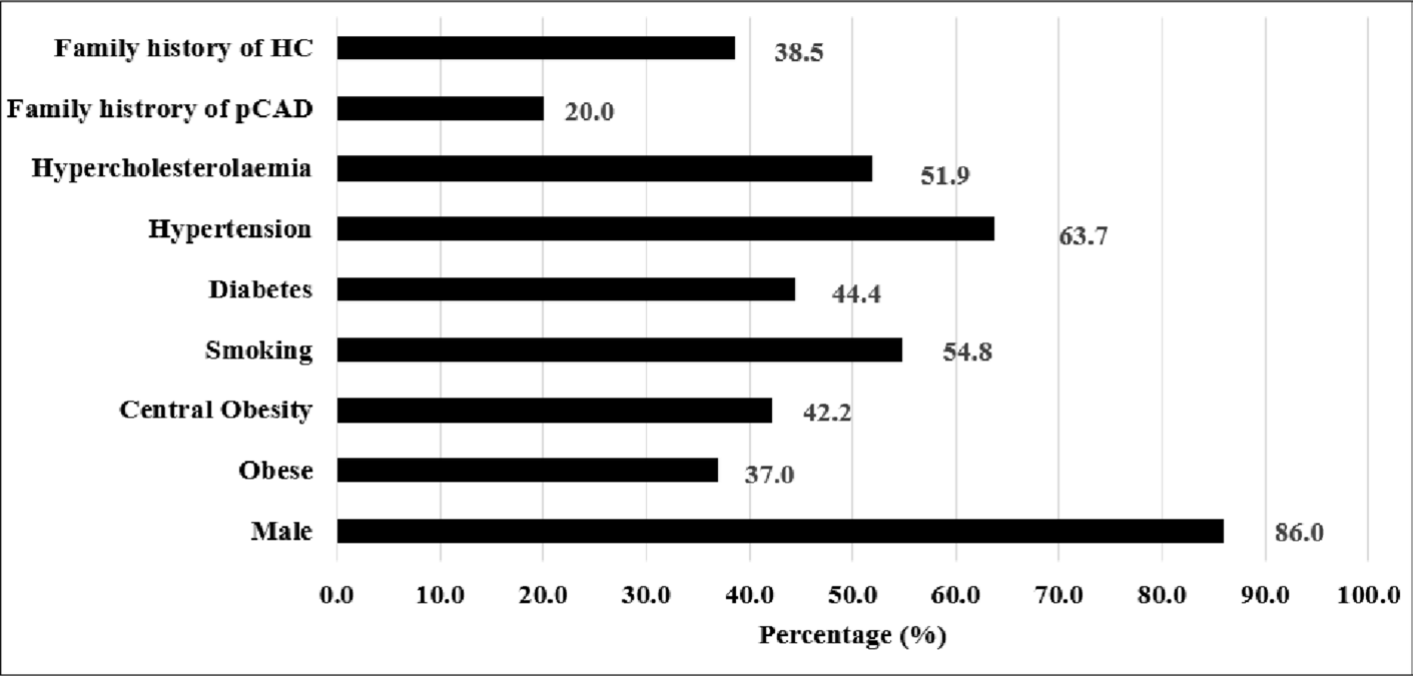
Conventional risk factors among individuals with pCAD (*n* = 135).

Patients with pCAD with and without FH from G1 (*n* = 70) and G2 (*n* = 65) were combined to determine the most common risk factors among pCAD patients. Figure 1 shows that after male gender (86.0%), smoking (54.8%), and hypertension (63.7%), HC (51.9%) was the most common risk factor in this cohort, followed by diabetes (44.4%), central obesity (42.2%), family history of HC (38.5%) and family history of pCAD (20.0%) (Fig. 1.)

### Correlation between Lp(a) and PCSK9

The correlation between Lp(a) and PCSK9 in each group was determined using Spearman’s rank correlation analysis. No statistically significant correlation between Lp(a) and PCSK9 was found in the pCAD group (G1 or G2). However, a weak correlation (*r* < 1) was found in G3 (*r* = 0.366, *p* = 0.019) and the combination of G1 and G2 (G1 + G2) (*r* = 0.366, *p* = 0.002) (*p* < 0.01, *p* < 0.05) (Table 4).

**Table 4:** The correlation between Lp(a) and PCSK9.

GROUP		PCSK9 (ng/ml)	
		r	p-value*
G1	Lp(a) (mmol/L)	0.236	0.102
G2		0.257	0.071
G3		0.366	0.019
G1 + G2		0.566	0.002
G1 + G2 + G3		0.051	0.532

*Spearman Correlation.

Abbreviation: G1 (pCAD + FH+), G2 (pCAD + FH-), G3 (CAD- FH-), G1 + G2 (pCAD + FH + and pCAD + FH-), G1 + G2 + G3 (pCAD + FH+, pCAD + FH-, and CAD- FH-).

### Association between PCSK9 and Lp(a) levels

**Fig. 2:**
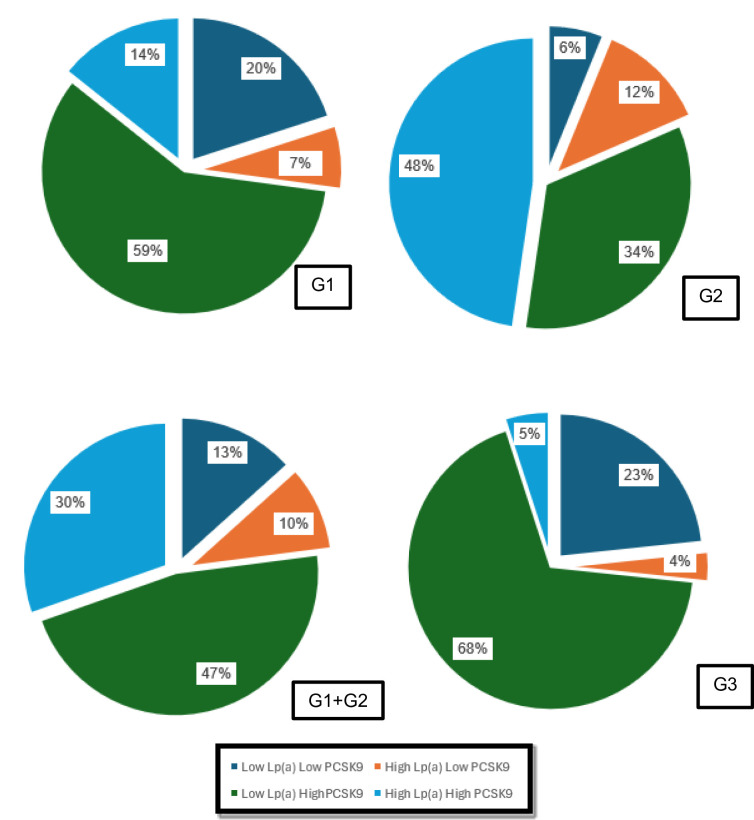
The Association between Lp(a) and PCSK9 level. Data are presented in percentage for each group following the category. Abbreviation: G1 (pCAD + FH+), G2 (pCAD + FH-), G1 + G2 (pCAD + FH + and pCAD + FH-), and G3 (CAD- FH-).

The association between Lp(a) and PCSK9 in each group was determined using Chi-Square analysis. No statistically significant correlation between Lp(a) and PCSK9 was found in pCAD (G1 or G2), all pCAD as well normal control group (G3) (*p* > 0.05). The distribution of patients into high and low Lp(a) following < 80th percentile (~ 50 mg/dl) for normal Lp(a)^48^, while high and low PCSK9 following the normal range of PCSK9 (170–220 ng/ml)^49^ (Fig. 2.).

### Simple logistic regression

The Lp(a), PCSK9, and CRFs associated with FH among pCAD patients were analysed using simple logistic regression. All data relating to the Lp(a), PCSK9, and the CRFs, including the lipid profiles, were included (Tables 5, 6 and 7).

**Table 5:** Simple logistic regression for G1 (pCAD + FH+).

Variable	Crude OR (95% CI)	Wald statistic (df)	*p*-value*
Lp(a) (mmol/l)	0.991 (0.991, 1.002)	1.893 (1)	0.169
PCSK9 (ng/ml)	1.000 (0.999, 1.000)	3.640 (1)	0.056
Age (years)	0.938 (0.962, 1.592)	0.078 (1)	0.781
Gender (Male/Female)	0.646 (0.302, 1.384)	1.263 (1)	0.261
Ethnicity (Malay/Chinese/Indian)	1.040 (0.557, 1.941)	0.015 (1)	0.903
BMI (Cont.)	2.388 (0.202, 5.747)	8.034 (1)	**0.005**
BMI (Normal/Overweight/Obese)	0.858 (0.3101, 1.094)	2.397 (1)	0.069
Waist Circumference (cm)	0.557 (0.2999, 1.038)	3.398 (1)	0.059
Central Obesity (Yes/No)	0.616 (0.311, 1.219)	1.938 (1)	0.054
Smoking (Yes/No)	3.838 (0.186, 5.616)	12.562 (1)	**0.000**
Diabetes (Yes/No)	1.398 (0.218, 2.726)	9.040 (1)	**0.003**
Hypertension (Yes/No)	2.062 (0.022, 3.170)	28.937 (1)	**0.000**
Family history of pCAD (Yes/No)	3.974 (0.091, 5.334)	27.697 (1)	**0.000**
Lipid Profiles			
Pre-treatment LDL-c (mmol/l) **^1^	2.378 (1.287, 5.678)	4.123 (1)	**0.031**
TC (mmol/l)	3.001 (0.782, 1.281)	3.001 (1)	**0.049**
TG (mmol/l)	1.740 (0.546, 1.003)	3.765 (1)	**0.048**
HDL-c (mmol/l)	4.436 (1.548, 12.717)	7.688 (1)	0.246

**Representing baseline LDL-c level prior to lipid-lowering medication and LDL-c level for drug-naïve individuals. ^1^ Several patients do not have the exact baseline lipid profile – statin conversion was used to calculate the estimated post-treatment LDL-c level.

Abbreviation: HC (hypercholesterolemia), Cont. (Continuous Data), BMI (body mass index), LDL-c (low-density lipoprotein cholesterol), TC (total cholesterol), TG (triglyceride), HDL-c (high-density lipoprotein cholesterol).

**Table 6:** Simple logistic regression for G2 (pCAD + FH-).

Variable	Crude OR (95% CI)	Wald statistic (df)	*p*-value*
Lp(a) (mmol/l)	3.991 (0.991,6.005)	2.898 (1)	0.789
PCSK9 (ng/ml)	5.001 (0.999, 11.002)	3.640 (1)	0.561
Age (years)	1.017 (0.962, 3.054)	2.078 (1)	0.581
Gender (Male/Female)	0.648 (0.302, 1.396)	1.263 (1)	0.221
Ethnicity (Malay/Chinese/Indian)	2.040 (0.557, 5.941)	0.015 (1)	0.303
BMI (Cont.)	2.824 (0.860, 3.869)	2.451 (1)	0.510
BMI (Normal/Overweight/Obese)	1.323 (0.950, 1.843)	1.610 (1)	0.178
Waist Circumference (cm)	3.457 (0.2999, 4.569)	2.952 (1)	0.081
Central Obesity (Yes/No)	0.457 (0.2999, 2.031)	2.451 (1)	0.065
Smoking (Yes/No)	1.111 (0.311, 1.219)	1.538 (1)	0.064
Diabetes (Yes/No)	2.190 (1.075, 6.238)	5.495 (1)	**0.019**
Hypertension (Yes/No)	0.398 (0.613, 6.726)	1.925 (1)	0.262
Family history of pCAD (Yes/No)	0.125 (0.004, 4.000)	1.384 (1)	0.239
Family history of HC(Yes/No)	1.246 (0.104, 14.892)	1.266 (1)	0.862
Lipid Profiles			
Pre-treatment LDL-c (mmol/l) **^1^	1.455 (0.998, 4.001)	2.190 (1)	0.081
TC (mmol/l)	3.027 (1.003, 5.208)	12.005 (1)	**0.010**
TG (mmol/l)	1.230 (1.286, 7.793)	5.456(1)	**0.049**
HDL-c (mmol/l)	1.01 (8.35, 40.717)	3.610 (1)	0.557

**Representing baseline LDL-c level prior to lipid-lowering medication and LDL-c level for drug-naïve individuals. ^1^ Several patients do not have the exact baseline lipid profile – statin conversion was used to calculate the estimated post-treatment LDL-c level.

Abbreviation: Abbreviation: HC (hypercholesterolemia), Cont. (Continuous Data), BMI (body mass index), LDL-c (low-density lipoprotein cholesterol), TC (total cholesterol), TG (triglyceride), HDL-c (high-density lipoprotein cholesterol).

**Table 7:** Simple logistic regression for G1 + G2 (pCAD + FH + and pCAD + FH-).

Variable	Crude OR (95% CI)	Wald statistic (df)	*p*-value*
Lp(a) (mmol/l)	3.032 (1.017,1.047)	17.196 (1)	0.000
PCSK9 (ng/ml)	1.000 (0.999, 3.000)	3.880 (1)	0.049
Age (years)	1.040 (0.993,1.090)	2.760 (1)	0.097
Gender (Male/Female)	0.350 (0.174, 1.705)	8.615 (1)	0.256
Ethnicity (Malay/Chinese/Indian)	0.513 (0.277, 0.949)	4.528 (1)	0.233
BMI (Cont.)	2.019 (0.520, 3.997)	1.222 (1)	0.389
BMI (Normal/Overweight/Obese)	0.563 (0.265, 1.196)	2.233 (1)	0.285
Waist Circumference (cm)	1.633 (0.875, 3.048)	2.377 (1)	0.432
Central Obesity (Yes/No)	1.923 (1.025, 3.607)	4.152 (1)	0.042
Smoking (Yes/No)	1.407 (0.199, 4.834)	6.027 (1)	0.014
Diabetes (Yes/No)	5.049 (0.017, 6.143)	30.697 (1)	0.000
Hypertension (Yes/No)	4.201 (0.106, 8.382)	24.00 (1)	0.000
Family history of pCAD (Yes/No)	1.313 (0.115, 0.852)	5.165 (1)	0.023
Family history of HC(Yes/No)	3.239 (0.110, 5.523)	12.848 (1)	0.000
Lipid Profiles			
Pre-treatment LDL-c (mmol/l) **^1^	3.526 (2.210, 5.627)	27.926 (1)	0.000
TC (mmol/l)	2.310 (0.218, 4.442)	41.873 (1)	0.000
TG (mmol/l)	1.521 (0.381, 3.713)	16.627 (1)	0.000
HDL-c (mmol/l)	0.940 (1.281, 12.113)	5.723 (1)	0.238

**Representing baseline LDL-c level prior to lipid-lowering medication and LDL-c level for drug-naïve individuals. ^1^ Several patients do not have the exact baseline lipid profile – statin conversion was used to calculate the estimated post-treatment LDL-c level.

Abbreviation: HC (hypercholesterolemia), Cont. (Continuous Data), BMI (body mass index), LDL-c (low-density lipoprotein cholesterol), TC (total cholesterol), TG (triglyceride), HDL-c (high-density lipoprotein cholesterol).

### Multiple logistic regression

Based on the simple logistic regression results, the independent variables with a p-value < 0.25 were included for further analysis using multiple logistic regression. In pCAD with clinically diagnosed FH, the significant values were hypertension (OR: 15.7), followed by BMI (OR: 9.2), pre-treatment LDL-c (OR: 9.0), and family history of HC (OR: 6.0) (*p* < 0.05). While, in pCAD + FH-, there were fewer independent predictors such as diabetes (OR: 2.4), followed by TC (OR: 1.5), and TG (OR: 1.4). Finally, the independent predictor for pCAD + with and without FH were pre-treatment LDL-c (OR: 6.5), followed by Lp(a) (OR: 5.0), TG (OR: 4.8), diabetes (OR: 3.0), smoking (OR: 2.1), hypertension (OR: 1.6), and TC (OR: 1.027) (Tables 8, 9 and 10).

**Table 8:** Multiple logistic regression for G1 (pCAD + FH+).

Variable	Adjusted OR (95% CI)	Wald statistic (df)	*p*-value*
Lp(a) (mmol/l)	0.993 (0.984, 1.003)	2.022 (1)	0.155
PCSK9 (ng/ml)	0.999 (0.998, 1.001)	1.081 (1)	0.299
BMI (Normal/Overweight/Obese)	9.231 (1.723, 11.256)	5.964 (1)	0.023
Waist Circumference (cm)	5.803 (0.291, 115.533)	1.328 (1)	0.249
Central Obesity (Yes/No)	2.539 (0.143, 45.034)	0.403 (1)	0.525
Smoking (Yes/No)	0.070 (0.03, 1.838)	2.542 (1)	0.111
Diabetes (Yes/No)	0.417 (0.065, 2.653)	0.859 (1)	0.354
Hypertension (Yes/No)	15.723 (1.723, 43.487)	5.964 (1)	0.001
Family history of HC(Yes/No)	6.028 (0.03, 8.282)	9.207 (1)	0.002
Lipid Profiles			
Pre-treatment LDL-c (mmol/l) **^1^	8.968 (2.393, 33.602)	10.593 (1)	0.045
TG (mmol/l)	0.408 (0.110, 1.509)	1.805 (1)	0.179
HDL-c (mmol/l)	1.758 (0.082, 37.793)	0.130 (1)	0.718

**Representing baseline LDL-c level prior to lipid-lowering medication and LDL-c level for drug-naïve individuals. ^1^ Several patients do not have the exact baseline lipid profile – statin conversion was used to calculate the estimated post-treatment LDL-c level.

Abbreviation: HC (hypercholesterolemia), Cont. (Continuous Data), BMI (body mass index), LDL-c (low-density lipoprotein cholesterol), TC (total cholesterol), TG (triglyceride), HDL-c (high-density lipoprotein cholesterol).

**Table 9:** Multiple logistic regression for G2 (pCAD + FH-).

Variable	Adjusted OR (95% CI)	Wald statistic (df)	*p*-value*
Central Obesity (Yes/No)	2.539 (0.143,45.034)	0.403 (1)	0.525
Smoking (Yes/No)	0.070 (0.03,1.838)	2.542 (1)	0.111
Diabetes (Yes/No)	2.417 (0.065,2.653)	0.859 (1)	0.04
Lipid Profiles			
Pre-treatment LDL-C (mmol/l) **^1^	0.543 (2.393,13.612)	10.593 (1)	0.281
TC (mmol/l)	1.523 (1.871,5.287)	12.005 (1)	0.032
TG (mmol/l)	1.408 (0.110,1.509)	1.805 (1)	0.048

**Representing baseline LDL-c level prior to lipid-lowering medication and LDL-c level for drug-naïve individuals. ^1^ Several patients do not have the exact baseline lipid profile – statin conversion was used to calculate the estimated post-treatment LDL-c level.

Abbreviation: Abbreviation: HC (hypercholesterolemia), Cont. (Continuous Data), BMI (body mass index), LDL-c (low-density lipoprotein cholesterol), TC (total cholesterol), TG (triglyceride), HDL-c (high-density lipoprotein cholesterol).

**Table 10:** Multiple logistic regression for G1 + G2 (pCAD + FH + and pCAD + FH-).

Variable	Adjusted OR (95% CI)	Wald statistic (df)	*p*-value*
Lp(a) (mmol/l)	5.036 (1.011,7.062)	8.000 (1)	0.015
PCSK9 (ng/ml)	1.000 (0.999, 1.001)	0.102 (1)	0.750
Age (years)	1.110 (0.933,1.321)	1.378 (1)	0.240
BMI (Normal/Overweight/Obese)	2.792 (0.092, 84.680)	0.348 (1)	0.555
Central Obesity (Yes/No)	0.186 (0.003, 9.947)	0.685 (1)	0.408
Smoking (Yes/No)	2.054 (0.002, 3.868)	2.609 (1)	0.003
Diabetes (Yes/No)	3.015 (0.001, 4.440)	5.929 (1)	0.005
Hypertension (Yes/No)	1.562 (0.064, 3.916)	0.271 (1)	0.001
Family history of pCAD (Yes/No)	0.125 (0.004, 4.000)	1.384 (1)	0.239
Family history of HC(Yes/No)	1.246 (0.104, 14.892)	0.030 (1)	0.862
Lipid Profiles			
Pre-treatment LDL-c (mmol/l) **	6.526 (2.210, 7.627)	27.926 (1)	0.025
TC (mmol/l)	1.027 (0.003, 2.208)	12.005 (1)	0.041
TG (mmol/l)	4.784 (1.283, 17.793)	5.456 (1)	0.039
HDL (mmol/l)	24.399 (0.835, 30.58)	3.610 (1)	0.057

**Representing baseline LDL-c level prior to lipid-lowering medication and LDL-c level for drug-naïve individuals. ^1^ Several patients do not have the exact baseline lipid profile – statin conversion was used to calculate the estimated post-treatment LDL-c level.

Abbreviation: HC (hypercholesterolemia), Cont. (Continuous Data), BMI (body mass index), LDL-c (low-density lipoprotein cholesterol), TC (total cholesterol), TG (triglyceride), HDL-c (high-density lipoprotein cholesterol).

## Discussion

Lp(a) is a quantitative genetic trait in human plasma that is highly heritable, with plasma level being predominantly (90%) determined by variation in the Lp(a) gene on chromosome 6q26-27, which affects the number of KIV2 copies among individuals, and displays extreme variation both within and between populations^50,51^. CAD patients had a significantly higher level of Lp(a)^52^, which is increasingly recognised as the strongest known genetic risk factor for pCAD^10^. In consensus in our studies, the AP-pCAD subject showed the highest level of Lp(a) in the pCAD group [G1: 27.2 (13.2–72.2) mmol/l, G2: 34.7 (12.7–100.9.7.9) mmol/l] and the lowest in the normal control group [group 3: 7.5 (7.0–14.7.0.7) mmol/l].

While elevated Lp(a) is well recognised as a genetic risk factor, accumulating evidence indicates that its contribution to ASCVD extends beyond circulating lipid levels that involve direct arterial wall injury. Lp(a) promotes endothelial dysfunction, vascular inflammation, oxidative stress, and plaque instability through pro-inflammatory and pro-thrombotic mechanisms, thereby accelerating atherogenesis at the level of the arterial intima^53–55^. These pathogenic effects are particularly relevant in FH patients, in whom elevated Lp(a) substantially contributes to premature and residual cardiovascular risk despite aggressive LDL-c lowering^56,57^.

In addition to its pathogenic role, Lp(a) has gained increasing importance in cardiovascular risk stratification. Notably, the recent focused update of the 2019 ESC/EAS Guidelines on the management of dyslipidaemias has recognised Lp(a) as a cardiovascular risk modifier, supporting its use to refine risk assessment and potentially reclassify individuals at intermediate cardiovascular risk into higher-risk categories^58^. This guideline update underscores the clinical relevance of our finding that Lp(a) independently predicts pCAD, particularly in younger individuals and those with FH, where traditional risk assessment tools may underestimate lifetime cardiovascular risk.

PCSK9 variants have variable frequencies in different populations and their impact on cholesterol levels^22,59–61^. More than 40 amino acid variants of PCSK9 have been shown to affect plasma cholesterol levels in humans^23,62,63^. These changes are classified as GOF mutations associated with high levels of LDL-c and LOF mutations associated with low LDL-c. GOF mutations result in mild to severe HC^22^. While Tada et al.^64^ recently reported that Lp(a) was elevated in patients with FH caused by PCSK9 GOF mutations to the same level as that in FH caused by LDLR mutations. PCSK9 variants were not directly assessed in this study. Our study focused on serum PCSK9 levels, rather than genetic variants. However, our research group has previously detected several pathogenic PCSK9 variants in the Malaysian population^22^, and it is possible that these variants could be present in this cohort. Almontashiri et al.^25^ found that plasma PCSK9 is elevated with acute MI in non-diabetic AP-pCAD without statin therapy. In our study, AP-pCAD patients showed a higher level of PCSK9 in both pCAD groups [G1: 4.31.4 (178.0–1008.0.0.0) ng/ml and G2: 471.4 (333.1–1188.0) ng/ml] and the lowest in the normal control group [group 3: 38.9 (147.1–566.2.1.2) ng/ml]. Beyond its established role in LDLR degradation^65^, PCSK9 has also been implicated in lipid-independent pro-atherogenic processes, including endothelial activation and vascular inflammation, suggesting a direct role in arterial wall pathology^53,66^.

To understand the mechanism by which PCSK9 mAb reduces Lp(a) levels^67,68^, elucidation of the correlation between serum levels of PCSK9 and Lp(a) is required. A recent study by Nekaies et al.^31^ reported that PCSK9 correlated positively with Lp(a). In contrast, Yang et al. reported that PCSK9 is not associated with Lp(a) levels^69^. Both reported the correlation between Lp(a) and PCSK9 in DM patients; however, it was conducted in a different population. Therefore, the correlation between PCSK9 and Lp(a) levels remains controversial. Specifically, a recent mechanistic study demonstrated that PCSK9 inhibition may influence Lp(a) metabolism through LDLR–independent pathways, including effects on apo(a) synthesis, intracellular trafficking, and secretion, providing a biologically plausible explanation for the observed Lp(a)-lowering effects of PCSK9 inhibitors despite modest or inconsistent correlations between circulating PCSK9 and Lp(a) levels^70^.

The absence of a strong linear association between circulating PCSK9 and Lp(a) levels does not preclude their combined contribution to atherosclerotic disease. Emerging evidence suggests that Lp(a)-mediated arterial injury and PCSK9-driven vascular inflammation may act through partially overlapping but biologically distinct mechanisms at the arterial wall, rather than through a direct correlation in plasma concentrations^54,66^. This may be particularly relevant in FH, where lifelong exposure to atherogenic lipoproteins amplifies vascular damage independent of short-term biomarker correlations^56^. This study was the first to report the correlation between Lp(a) and PCSK9 in parallel in a cohort of pCAD with and without clinically diagnosed FH. There was no significant correlation between Lp(a) and PCSK9 in either G1 (pCAD + FH-) or G2 (pCAD + FH-). This lack of correlation is due to (i) the variability in Lp(a) and PCSK9 concentrations among many patients, which can be extremely high or low (Table 3), and (ii) the inconsistency in the relationship between Lp(a) and PCSK9 across patients. This inconsistency is likely influenced by statin medication, as PCSK9 concentrations were measured while patients were on statins. No specific types or dosages of statins were included as criteria for this study. Studies have reported that PCSK9 concentrations increase with statin therapy^71,72^. Statins reduce hepatic intracellular cholesterol, leading to an increase in LDL receptors (LDLRs) and PCSK9 protein levels^71,72^. Although there is no clear dose-response effect of statin treatment on PCSK9 levels, higher doses of statins tend to increase circulating PCSK9 levels. Welder et al.^73^ reported high-dose of atorvastatin cause a rapid, sustained increase in human serum PCSK9 and disrupts its correlation with LDL cholesterol. Thus, we postulated that different dosages and types of statins may also affect PCSK9 concentrations. The unknown effects of statins on PCSK9 might contribute to the lack of association between Lp(a) and PCSK9 in these two groups. However, a weak correlation (*r* < 1.0) between Lp(a) and PCSK9 concentrations was observed when G1 and G2 were combined into a single group [pCAD with and without FH (G1 and G2)]. This could be due to the larger sample size and the matching of inconsistent data when the groups were combined. Moreover, genetic testing for common mutations such as LDLR, apoB, and PCSK9 was not performed in this study, so the classification of patients into FH and non-FH was based solely on clinical diagnosis. When G1 and G2 were combined, it is possible that all FH patients were grouped, correlating with one another. The NC group (G3), which was age and gender-matched with the pCAD group, also showed a weak correlation (*p* < 0.05). G3 represents the normal control group, consisting of patients not on statins. Therefore, a significant correlation could be established.

Our findings suggest that Lp(a) interacts with PCSK9 in these two groups, indicating the vital role of Lp(a) and PCSK9 in predicting CAD regardless of the clinically diagnosed FH. Variations in terms of correlation between Lp(a) and PCSK9 observed between patients may also be attributed to differences in race, as both Lp(a) and PCSK9 concentration vary considerably by race and other factors^29^. The current cutoff for Lp(a) is not appropriate and suitable for all individuals. As reported by Guan et al.^74^, for Caucasian and Hispanic individuals, the Lp(a) cutoff above 50 mg/dl should be considered, while for Black individuals, the 30 mg/dl cutoff remains. The Lp(a) cutoff needs to be race-specific^75^. This study was the first to report on Lp(a) and PCSK9 in parallel in a cohort of pCAD with and without clinically diagnosed FH among three different races in Malaysia.

Lp(a) is a hepatically synthesised particle resembling LDL, comprising an apoB100 molecule connected to a significantly large glycoprotein called apo(a)^34,38^. The biological role of Lp(a) remains unknown; yet an increase in Lp(a) concentration has been identified as an independent risk factor for ASCVD^9^. Several recent clinical trials have provided evidence that PCSK9 antibodies are a promising novel candidate drug for lowering LDL-c and Lp(a)^76,77^. Nevertheless, the mechanism by which PCSK9 inhibitors reduce Lp(a) levels remains unclear.

In this cross-sectional study, Lp (a) and PCSK9 concentrations were highest in the G2 group (pCAD + FH-) and lowest in the G3 (NC group). However, no association was found between Lp(a) and PCSK9 in all groups. According to Yang et al.^69^, non-significant variations in Lp(a) and PCSK9 levels were discovered among T2DM patients who had greater percentages of hypertension, hyperlipidaemia, and CAD compared to non-T2DM patients. In addition, they found no statistically significant relationship between PCSK9 and Lp(a) in their study sample. In line with their finding, our study also found no association between PCSK9 level and Lp(a) concentration in the pCAD patient with a higher percentage of diabetes mellitus (DM) (55%). Contrary to our findings, Nozue et al.^70^ reported that heterodimer PCSK9 levels were positively correlated with Lp(a) concentration. These inconsistent results may be attributed to the different forms of PCSK9 being measured. Our study measured all forms of PCSK9, active and not active PCSK9 altogether. However, Nozue et al.^78^ measured the active form (heterodimer PCSK9) and non-active form (furin-cleaved PCSK9) separately. Heterodimer PCSK9 was considered the form with a stronger binding to and degradation of LDLR^79^ and furin-cleaved heterodimer with reduced affinity for LDLR^80^. The connection between Lp(a) and furin-cleaved PCSK9 was insignificant in their research. The measurement of PCSK9 levels as a whole may contribute to the weak or no association between PCSK9 and Lp(a). Furthermore, based on Table 2, the ethnicity was significantly different between each group (*p* < 0.05). This may contribute to the no correlation, as a previous study reported Lp(a) and PCSK9 concentration vary considerably by race^29^.

Coronary risk factors such as obesity^45,81,82^, type 2 diabetes mellitus (T2DM)^82^, TC^83^, and LDL-c^84^ play a role in contributing to the high PCSK9 levels. Numerous current findings also suggest that circulating PCSK9 concentrations are associated with the incidence of CAD^24,85,86^. In line with our study, the PCSK9 was highest in the pCAD group, with the highest proportion of obesity, hypertension, and HC subjects with the highest TC, TG, and LDL-c. Traditional predictors of CAD risk, such as age, gender, smoking, hypertension, DM, family history of CAD, HDL-c, TG, LDL-c, and Lp(a) levels, have been studied and reported as potential predictors of atherosclerotic burden and/or CVD prognosis among people with FH^87,88^. In our study, the simple logistic regression was performed to determine the significant predictors of pCAD. All related data, such as Lp(a), PCSK9, and CRFs, including the lipid profile, were included in the analysis.

Furthermore, other considerations may also explain the disagreement in the result. First, a comparison of Lp(a) concentration distributions in different ethnic groups reveals significant inter-racial variances. Variations in PCSK9 and Lp(a) correlation between patients may be related to racial differences, as PCSK9 and Lp(a) levels vary significantly by race and other factors. Second, our study includes three major races in Malaysia, which may contribute to the varying amounts of Lp(a) within the study. Third, the measurement of Lp(a) concentration is not standardised, and Lp(a) concentration may be influenced by LDL-c concentration. This could be the difficulty in establishing the link between Lp(a) and PCSK9 in our work. Future studies will investigate this possibility by exploring the role of PCSK9 variants in this population. The measurement of PCSK9 levels as a whole, rather than specific variants, may contribute to the weak or absent association between PCSK9 and Lp(a).

Our study provides new insights into the relationship between Lp(a) and PCSK9 levels in an Asian cohort, which may diverge from findings in other populations. This contributes to the current understanding, particularly among ethnic groups that are underrepresented in existing research. Moreover, our study underscores the critical importance of distinguishing between (i) gain- and loss-of-function mutations in PCSK9 and (ii) measuring the concentrations of active PCSK9 (mature-PCSK9) versus inactive PCSK9 (furin cleaved-PCSK9), rather than total PCSK9, as this differentiation likely influences the accuracy of associations observed in our findings.

## Conclusion

Lp(a) emerged as an independent predictor of premature coronary artery disease (pCAD), while its relationship with PCSK9 appeared modest and context dependent. Although PCSK9 showed a weak correlation with Lp(a) levels, most notably within the normal control group, this cross-sectional study cannot establish causality. These findings suggest a more complex interplay between Lp(a) and PCSK9, potentially influenced by factors such as lipid-lowering therapy. Further prospective and mechanistic studies are warranted to clarify the biological pathways linking these biomarkers and to evaluate the clinical utility of PCSK9 in cardiovascular risk stratification, particularly in high-risk populations.

### Limitations

There are several limitations to our study. First, the study design was cross-sectional. Second, the blood samples collected for PCSK9 measurement were not from statin-naive pCAD patients, as the patients were angiogram-proven-pCAD patients, where statin administration is a standard management for pCAD. Although statin administration will decrease the cellular synthesis of LDL-c, which triggers the homeostatic response of LDLR upregulation, the PCSK9 will also be upregulated. Therefore, there is the possibility that the PCSK9 levels measured in this study were higher than usual. Third, three races were present in the study, which might contribute to the higher variation. Finally, the small number of patients included in the study resulted in low statistical power.

## Data Availability

The datasets used and/or analysed during the current study are available from the corresponding author on reasonable request.
